# Subclinical Neuropathy in Children With Type I Diabetes Mellitus: Tertiary Care Centre Experience

**DOI:** 10.7759/cureus.27765

**Published:** 2022-08-08

**Authors:** Waleed A Altuwaijri, Angham N Almutair, Ibrahim A AlAlwan, Maria J Almahdi, Sulaiman D Almasoud

**Affiliations:** 1 Department of Pediatrics, Division of Neurology, King Abdulaziz Medical City Riyadh, Riyadh, SAU; 2 Department of Pediatrics, Division of Endocrinology, King Abdulaziz Medical City Riyadh, Riyadh, SAU

**Keywords:** pediatric neuropathy, nerve conduction study (ncs), diabetic peripheral neuropathy (dpn), subclinical neuropathy, type i diabetes mellitus

## Abstract

Introduction: Diabetic peripheral neuropathy is a common complication of diabetes mellitus (DM) type 1. However, it can occur without evidence of symptoms or clinical signs of neuropathy labeled as subclinical neuropathy, which neurophysiological studies can best detect.

Purpose: To evaluate the prevalence of subclinical neuropathy among children with DM type 1, determine the association with blood sugar control, and evaluate the pattern of nerve involvement in neurophysiological studies.

Methods: This cross-sectional study evaluated 100 children with DM type 1, aged five to 15 years, at least one year after the diagnosis. Subclinical neuropathy was evaluated using nerve conduction study. Glycemic control was assessed using hemoglobin A1c (HbA1c).

Results: The mean age of subjects was 11.5 ± 0.25 years. The average age at the onset of the disease was 5.95 ± 0.25 years. There were 64 patients who had electrophysiological evidence of peripheral neuropathy. The most observed electrophysiological changes were distal latency abnormalities in the left and right peroneal nerves in 39 and 33 patients, respectively. Sensory nerve amplitude, peak latency, and conduction velocity were normal in all patients (100%). HbA1c level did not show a statistically significant association with the incidence of subclinical neuropathy.

Conclusion: Subclinical neuropathy was prevalent in children with DM type 1. Poor glucose control was only associated with an increased odds ratio of subclinical neuropathy.

## Introduction

Subclinical neuropathy is defined as asymptomatic peripheral neuropathy, which is one of the common complications of patients with diabetes mellitus (DM). There is considerable uncertainty about the prevalence of diabetic peripheral neuropathy (DPN) in pediatric populations, which is probably due to the lack of large epidemiological studies performed on pediatric patients with DM, who often show few symptoms of neurological involvement [1]. The reported prevalence of diabetes-related peripheral neuropathy ranges from 16% to as much as 87%. Less than one-third of physicians recognize the manifestations of diabetes-related peripheral neuropathy, even when the patient is symptomatic [2]. One possible explanation for this wide difference in prevalence is that peripheral neuropathy tends to be subclinical [3].

Many risk factors have been suggested for peripheral neuropathy, such as the duration of diabetes, age at onset, height, puberty, positive family history, and diabetic complications of puberty [4]. Hajas et al. studied the effect of disease duration on the development of DPN and followed up with patients at one, five, and 10 years after diagnosis [5]. The study showed a strong association of the duration of diabetes with subclinical neuropathy, with rates ranging from 17.7% at one year after diagnosis to 46.8% after 10 years. However, hyperglycemia has been the single most important risk factor studied in the development of diabetic complications [6,7].

The effect of chronic hyperglycemia is mediated by the increase of the intracellular level of glucose and sorbitol, which eventually leads to oxidative stress and the generation of free radicals, which damage intracellular macromolecules. In addition, chronic hyperglycemia induces the formation of advanced glycation end products (AGEs), which modify the structure of myelin, leading to axonal degeneration [4].

Although there are no guidelines on screening subclinical diabetic neuropathy, nerve conduction study (NCS) is the most common tool used for diagnosing it [8]. Some studies have looked at the sensitivity of NCS compared to clinical examination or non-invasive testing, which showed that NCS had better specificity and sensitivity [8,9].

The pattern of nerve dysfunction has varied in neurophysiological studies on pediatric populations [10]. It was thought that sensory involvement is the earliest NCS sign in children, similar to adult diabetic patients, but other studies found motor involvement to be the earliest sign of neuropathy in pediatric patients [10-12].

This study evaluated the prevalence of subclinical neuropathy among Saudi children and adolescents diagnosed with DM type 1 using a nerve conduction study. We also aimed to observe the effect of the blood sugar level, age at diagnosis, and disease duration on the development of subclinical neuropathy. The secondary objective was to study the electrophysiological pattern of nerve involvement.

## Materials and methods

The study was conducted in King Abdulaziz Medical City in Riyadh. We examined 100 patients (46 males and 54 females) aged five to 15 years (mean 11.5 ± 0.25 years) who were diagnosed with DM type 1 and underwent neurophysiological testing at least one year after the diagnosis. Patients routinely followed up at the pediatric clinic of King Abdulaziz Medical City were invited to participate in the study. All patients diagnosed with DM type 1 and treated with multiple daily injections or insulin pumps were included. Patients were excluded if they had diabetic ketoacidosis, blood sugar levels less than 5 mmol/L or more than 15 mmol/L before NCS, neurological or metabolic diseases, or if they were uncooperative.

All patients’ age, duration of diabetes, and other demographic characteristics were documented. Each patient underwent neurological examination by a neurologist, who examined muscle power, fine touch, pinprick, pain, temperature, vibration, proprioception, and deep tendon reflexes. All patients underwent NCS in the pediatric ambulatory clinic. Hemoglobin A1c (HbA1c) and fasting blood sugar were obtained before the visit.

Approval for this study was obtained from the ethics committee of King Abdullah International Medical Research (approval # RC10-093), and written informed consent was obtained from the guardians of patients.

Nerve conduction study

NCS was performed using a standard technique with surface electrodes [13]. The variables recorded included the motor conduction velocities (CVs), amplitudes of compound muscle action potential (CMAP), and distal latency (DL) in the median, ulnar, posterior tibial, and peroneal nerves. In addition to CV, amplitudes of sensory nerve action potential (SNAP) and peak latency in the median, ulnar, and sural nerves were measured. All recordings were performed by the same experienced technician and interpreted by a pediatric neurologist. The criterion for abnormal values of these tests was values beyond the mean ± 2.5 standard deviations of normal values [14]. At least two abnormal independent neurophysiological nerve parameters were required as criteria for peripheral nervous system involvement.

Statistical analysis

All statistical analyses were done using SPSS software (IBM Corp., Armonk, NY, USA). The percentage (%) of patients with abnormal nerve conduction and the specific nerves involved (median, ulnar, peroneal, tibial, and sural) were calculated. A chi-squared test was used to assess the association between the incidence of subclinical neuropathy and HbA1c, age at the time of test, and age at the time of diagnosis. Logistic regression analysis was used to assess predictors of subclinical neuropathy. Age, gender, type of insulin, HbA1c, and physical examination were included as independent variables. All data were expressed as the mean ± standard deviation, and a p-value < 0.05 was considered significant.

## Results

The study sample included 100 respondents (45% males and 55% females). The average age of the included patients was 11.5 ± 2.25 years with an average age at diagnosis of 5.95 ± 2.5 years, and the average duration of the disease was 5.5 ± 2.65 years. The average HbA1c was 10.1 ± 1.7. Regular insulin was used by 70% of the patients, and an insulin pump was used by 30%. The NCS result was normal in 36% of patients and abnormal in the remaining 64%. Physical examination was normal in 90% of the patients, as shown in Table 1.

**Table 1:** Descriptive statistics for the study sample. MDI: Multiple daily injections, HbA1c: Hemoglobin A1c

	[All]	N
	N=100	
Gender:		100
Male	45 (45%)	
Female	55 (55%)	
Age	11.5 (± 2.31)	100
Age at diagnosis	5.93 (± 2.82)	100
Duration of the disease	5.59 (± 2.63)	100
HbA1c	10.1 (± 1.68)	100
Type of insulin:		100
MDI	70 (70.0%)	
Insulin pump	30 (30.0%)	
Nerve conduction study:		100
Normal	36 (36.0%)	
Abnormal	64 (64.0%)	
Physical examination:		100
Normal	90 (90.0%)	
Abnormal	10 (10.0%)	

Subclinical neuropathy was present in 62.2% (n = 56) of patients. That is, 62.2% of the patients with normal physical examination had abnormal NCS results. The results showed that gender was not significantly associated with abnormal nerve conduction (OR = 0.65, p > 0.05). Age, age at diagnosis, and the disease's duration did not show a statistically significant association with an abnormal NCS. Higher A1c was associated with an abnormal NCS (OR = 1.25, p < 0.1), although the association was only significant at the level of 0.05. Physical examination abnormalities did not show a statistically significant association with abnormal NCS (Table 2).

**Table 2:** Factors associated with abnormal nerve conduction. MDI: Multiple daily injections, NCS: Nerve conduction study, HbA1c: Hemoglobin A1c

	NCS result			
	Normal	Abnormal	OR	P value
	N=36	N=64		
Gender:				0.384
Male	13 (37.1%)	31 (48.4%)	Ref.	
Female	22 (62.9%)	33 (51.6%)	0.63 [0.27;1.47]	
Age	11.1 (1.97)	11.8 (2.47)	1.13 [0.95;1.36]	0.145
Age at diagnosis	5.44 (2.53)	6.20 (2.96)	1.10 [0.95;1.28]	0.179
Duration of the disease	5.64 (2.83)	5.57 (2.53)	0.99 [0.85;1.16]	0.904
HbA1c	9.72 (1.57)	10.3 (1.72)	1.25 [0.97;1.62]	0.077
Type of insulin:				1.000
MDI	25 (69.4%)	45 (70.3%)	Ref.	
Insulin pump	11 (30.6%)	19 (29.7%)	0.96 [0.39;2.40]	
Physical examination:				0.323
Normal	34 (94.4%)	56 (87.5%)	Ref.	
Abnormal	2 (5.56%)	8 (12.5%)	2.29 [0.52;17.5]	

The HbA1c level did not show a statistically significant association with the incidence of subclinical neuropathy (p = 0.15), although the prevalence was higher in patients with HbA1c > 10%. The age at the time of diagnosis did not show a statistically significant association with the incidence of subclinical neuropathy (p = 0.125). The distribution of subclinical neuropathy was not significantly associated with age at diagnosis, although the prevalence of patients aged > 10 years was higher in patients with subclinical neuropathy (Table 3).

**Table 3:** Association between abnormal NCS and HbA1c, age and age at diagnosis. NCS: Nerve conduction study, HbA1c: Hemoglobin A1c

	NCS		P Value
	Normal	Abnormal	
HbA1c			0.171
7% - 8%	3 (42.9%)	4 (57.1%)	
8% - 9%	11 (55%)	9 (45%)	
9% - 10%	4 (22.2 %)	14 (77.8)	
> 10%	18 (32.7 %)	37 (67.3 %)	
Age at the time of test			0.128
10 years or less	13 (40.63%)	19 (59.38%)	
10 – 12 years	14 (43.75%)	18 (56.25%)	
> 12 years	8 (22.22%)	28 (77.78%)	
Age at diagnosis			0.727
< 5 years	14 (38.89%)	22 (61.11%)	
5 – 10 years	20 (33.9%)	39 (66.1%)	
> 10 years	1 (20%)	4 (80%)	

The abnormal NCS findings are presented in Table 4. The most significant finding was the early involvement of motor nerves in all patients with electrophysiological changes with no sensory nerve abnormalities. The most observed electrophysiological changes were DL abnormalities in the left and right peroneal nerves in 33% and 39% of the patients, followed by CV abnormalities in the left and right peroneal nerves in 27% and 31% of the patients, and CV abnormalities in the left and right tibial nerves in 37% and 36% of the patients, respectively. The amplitude of CMAP showed abnormality only in the median nerve in one patient (1%). Sensory CVs, peak latency, and amplitudes of SNAP were normal in all subjects (100%).

**Table 4:** Motor nerve conduction (MNC) data distribution. DL: Distal latency, CV: Conduction velocity

	Latency (DL)	Amplitude	Velocity (CV)
Nerve	N (%)	N (%)	N (%)
Median left			
Normal	88 (88.00)	100 (100.00)	98 (98.00)
Abnormal	12 (12.00)	0 (0.00)	2 (2.00)
Ulnar left			
Normal	100 (100.00)	100 (100.00)	100 (100.00)
Abnormal	0 (0.00)	0 (0.00)	0 (0.00)
Median right			
Normal	88 (88.00)	99 (99.00)	98 (98.00)
Abnormal	12 (12.00)	1 (1.00)	2 (2.00)
Ulnar right			
Normal	100 (100.00)	100 (100.00)	100 (100.00)
Abnormal	0 (0.00)	0 (0.00)	0 (0.00)
Peroneal left			
Normal	67 (67.00)	100 (100.00)	73 (73.00)
Abnormal	33 (33.00)	0 (0.00)	27 (27.00)
Tibial left			
Normal	91 (91.00)	100 (100.00)	63 (63.00)
Abnormal	9 (9.00)	0 (0.00)	37 (37.00)
Peroneal right			
Normal	61 (61.00)	100 (100.00)	69 (69.00)
Abnormal	39 (39.00)	0 (0.00)	31 (31.00)
Tibial right			
Normal	90 (90.00)	100 (100.00)	64 (64.00)
Abnormal	10 (10.00)	0 (0.00	36 (36.00)

## Discussion

DPN is a common complication of type 1 and type 2 DM and is often under-recognized and under-screened. In our study, we tried to look at subclinical neuropathy, asymptomatic patients with abnormal NCS. We found 64 patients out of 90 patients with normal physical examination with NCS changes related to DPN, and out of 64 patients with abnormal NCS, 58 patients (90%) were asymptomatic. This is in line with similar studies where NCS was used to diagnose DPN. The prevalence of DPN in our study was 64%, which is relatively high compared to other studies conducted with a larger sample size [15,16]. However, a very wide range of prevalence has been reported in the literature, probably due to the difference in the criteria applied to diagnose DPN.

The SEARCH study was a population-based prospective cohort study with a sample size of 1734, which found a DPN prevalence of 7% using the Michigan Neuropathy Screening Instrument [15]. On the other hand, a Danish nationwide study of 339 patients found a DPN prevalence of 62% using the Vibration Perception Threshold (VPT) [17]. Similar to our study, in a recent study on 156 patients, 55.1% of patients had subclinical neuropathy [18]. In the study, they divided the patients into four categories with possible and probable neuropathy among symptomatic patients and confirmed neuropathy for symptomatic patients with abnormal sural nerve conduction. When examining subclinical neuropathy for asymptomatic patients with abnormal sural nerve conduction, 80 of the 156 patients had abnormal sural nerve conduction, and only four were symptomatic. Similarly, Toopchizadeh et al. found that 57% of patients had DPN using electrophysiological studies [12]. The use of NCS might have increased the detection rate of DPN compared to other modalities, which explains the wide range of prevalence observed in the literature.

Several demographic and non-demographic predictors of increased risk of DPN have been studied in the literature. In our study, gender, age, age at diagnosis, duration of disease, abnormality on physical examination, type of insulin used, and HbA1c did not show any statistically significant association. Similarly, in a cross-sectional study, Hasani et al. did not find HbA1c and demographic factors to be correlated with DPN [3]. However, the duration of the disease showed a statistically significant association. Toopchizadeh et al. also did not find any significant association between abnormal NCS and age, gender duration of disease, and HbA1c [3].

Multiple studies have investigated HbA1c levels over a long period or glucose levels over time in relation to the outcome of NCS [1,5,19,20]. Hajas et al. showed that metabolic control using HbA1c over a 10-year follow-up period was significantly associated with the development of DPN [3]. Similarly, Lee et al. had similar results over a five-year follow-up period [1]. Pinto et al. retrospectively studied the relationship of abnormal NCS results with mean HbA1c and its variability over 10 years [19]. They found that the variability of HbA1c over 10 years was an independent risk factor. Although these findings of multiple risk factors for the outcome of NCS are conflicting, HbA1c level and metabolic control in longitudinal studies were the most significant predictors of the development of DPN. 

In our electrophysiological studies, motor nerves showed early involvement in peroneal and posterior tibial nerves with no sensory nerve abnormalities detected. The most observed abnormalities in our study were peroneal nerve and posterior tibial nerve CV and DL, which is similar to findings in other studies [1,21,22]. Schiller et al. conducted a study on 34 patients, including 17 patients diagnosed with DM type 1 and 17 normal controls [22]. The only NCS abnormality found was the slowing of MCV of the median nerve. Similarly, Hyllienmark et al. found that peroneal MCV was the most observed change followed by sural nerve SNAP [21]. In addition, they found out that a change in peroneal and median nerve CV and sural SNAP preceded clinical neuropathy by 13 years. In a five-year longitudinal study, Lee et al. found that the most affected nerves were peroneal, median motor, and sural nerves [1]. In addition, they observed a steady deterioration of the motor nerves over the course of five years. These findings collectively suggest that motor nerves are the first to be affected by DM type 1 in the pediatric age group and tend to be subclinical in most cases.

The use of NCS is a strength of our study as it is the most sensitive method to detect subclinical neuropathy. Although the use of NCS as a screening tool might sometimes increase the detection of minor changes that are unlikely to result in clinical neuropathy in the long term. The cross-sectional design and small sample size are limitations to our study. A longitudinal follow-up with a larger sample and a multi-center study of the nerve involvement in NCS and HbA1c level would yield a better picture of DPN in DM type 1 in the pediatric age group. A follow-up study after five years with neurological examination, HbA1c, and NCS will be carried out in the future with the same patients to follow up on the outcome of DPN.

## Conclusions

Peripheral neuropathy is one of the most common complications of DM. In the study, we examined the prevalence of subclinical neuropathy in patients with type 1 DM. The study shows high prevalence of subclinical neuropathy with use of NCS, probably due to sensitivity of NCS to minor changes. Demographic factors, duration of the disease and HbA1c did not show a statistically significant association.
